# Model Selection and Evaluation Based on Emerging Infectious Disease Data Sets including A/H1N1 and Ebola

**DOI:** 10.1155/2015/207105

**Published:** 2015-09-15

**Authors:** Wendi Liu, Sanyi Tang, Yanni Xiao

**Affiliations:** ^1^College of Mathematics and Information Science, Shaanxi Normal University, Xi'an 710062, China; ^2^Department of Applied Mathematics, Xi'an Jiaotong University, Xi'an 710049, China

## Abstract

The aim of the present study is to apply simple ODE models in the area of modeling the spread of emerging infectious diseases and show the importance of model selection in estimating parameters, the basic reproduction number, turning point, and final size. To quantify the plausibility of each model, given the data and the set of four models including Logistic, Gompertz, Rosenzweg, and Richards models, the Bayes factors are calculated and the precise estimates of the best fitted model parameters and key epidemic characteristics have been obtained. In particular, for Ebola the basic reproduction numbers are 1.3522 (95% CI (1.3506, 1.3537)), 1.2101 (95% CI (1.2084, 1.2119)), 3.0234 (95% CI (2.6063, 3.4881)), and 1.9018 (95% CI (1.8565, 1.9478)), the turning points are November 7,November 17, October 2, and November 3, 2014, and the final sizes until December 2015 are 25794 (95% CI (25630, 25958)), 3916 (95% CI (3865, 3967)), 9886 (95% CI (9740, 10031)), and 12633 (95% CI (12515, 12750)) for West Africa, Guinea, Liberia, and Sierra Leone, respectively. The main results confirm that model selection is crucial in evaluating and predicting the important quantities describing the emerging infectious diseases, and arbitrarily picking a model without any consideration of alternatives is problematic.

## 1. Introduction

Emerging and reemerging infectious diseases such as severe acute respiratory syndrome (SARS) in 2003 [1, 2], novel influenza (A/H1N1) pandemic in 2009 [3], and Ebola outbreak in West Africa in 2014 significantly affect public health, economic activity, and population movements. In particular, the 2014 Ebola outbreak in West Africa represents the largest outbreak of Ebola virus to date. Public health interventions have been introduced in all affected countries and show the great effects on the infection. However, the numbers of infected cases from Ebola show a trend of bouncing back after declining in February 2015. Those indicate that the outbreak patterns of Ebola in West Africa become more and more complex, and hence it is important to determine the best model by employing mathematical models and model selection methods, which can be used to estimate and predict the characteristics of emerging infectious diseases.

Although susceptible-infective-removal (SIR) compartmental model is commonly used to describe the transmission dynamics of an infectious disease, it cannot be used when we consider only the cumulative infected population and capture the temporal variations of an outbreak, such as the turning point that is the point in time at which the rate of accumulation changes from increasing to decreasing. Several models have been proposed to estimate basic reproduction number, turning point, and final size by cumulated cases; some of them are based on purely empirical relationship, while others have a theoretical basis and are realized by differential equations. The simplest and commonly applied model among all the infectious disease models is the Richards model [3–5]. By employing Richards model, Hsieh et al. investigated the characteristics including basic reproduction number, turning point, and final size for influenza such as H1N1 [3], SARS [4], and Dengue [5] by fitting Richards model to the reported cumulative cases.

The most common approach in infective disease data analyses with simply ODE model is to select one model, usually Richards model, based on the shape of the desired curve and on biological assumptions. A single wave of infections consisting of a single peak of high incidence, an S-shaped cumulative epidemic curve, and a single turning point of an outbreak can be the best fitting to data using the selected model. Inference and estimation of parameters and their precision are based on the fitted model. Therefore, the interesting questions would be as follows: Can Richards model effectively predict the growth of the cumulative infected population? How to select the best model for fitting the emerging infectious diseases data? Is it possible to predict the turning point and final size and effectively estimate the basic reproduction number which are quite important in the disease control and management?

The traditional approaches of hypotheses testing, when applied to model selection, have been often found to be mediocre [6, 7]. The adjusted coefficient of multiple determination that is often used in model selection was found to be a very poor approach [8]. Posada and Buckley [9] pointed out that the Bayesian and Akaikes information criterion (AIC) approaches present several important advantages over other model selection methods. Therefore, in the present work we employ the Bayes factors to select one model from a set of competing models which can capture the underlying disease outbreak best, and further it can be confirmed by calculating AIC values. The basis of the Bayes factor approach to model selection is quantifying the plausibility of each model when the data and the set of candidate models are given. The Bayes factor is a measure of the change from prior model odds to posterior model odds, brought about by the observed data. In this study, we calculate the Bayes factor with the ratio of the selected number of different models and sample from the joint space of product of model and parameters in each model and then estimate the posterior probability of each model using Metropolis-Hastings (MH) algorithms.

In Section 2 we initially present the data sources and the important quantities describing the emerging infectious diseases for this study and then briefly provide the approaches of Bayesian model selection and realization algorithms. In Section 3 we verify the validity of the model selection algorithm introduced in Section 2. In Section 4 based on the real data sets for 2009 A/H1N1 in Shaanxi Province of mainland China and the data sets for current Ebola infection in West Africa, we select the optimal model and examine the specifics of the corresponding diseases. In particular, we focus on estimating basic reproduction number, turning point, and final size of A/H1N1 and Ebola and then explain some important issues related to the emerging infection disease control. Finally, we conclude by summarizing important conclusions and emphasizing the importance of model selection.

## 2. Data and Methods

### 2.1. Data Sources, Basic Reproduction Number, Turning Point, and Final Size

We employ the data on laboratory-confirmed cases of pandemic A/H1N1 influenza admitted to the 8th Hospital of Xi'an, the Province's Public Health Information System [11, 13], in 2009. The data included information on the daily number of hospital notifications and the number of newly reported hospital notifications (local/imported cases in mainland China or community/sporadic cases in Shaanxi Province). For the Ebola data sets, we use the data from the WHO website for the most serious regions including Guinea, Liberia, and Sierra Leone from March 25, 2014, to May 3, 2015. Note that the data for Ebola are the sum of confirmed, probable, and suspected cases.

As mentioned in Section 1, the main purpose is to choose the best model from the several single species growth models, which will help us to evaluate the characteristics of emerging infectious diseases including A/H1N1 and Ebola. In particular, the basic reproduction number, turning point, and final size are the most important quantities describing the emerging infectious diseases. Thus, we first estimate the parameter values for each model candidate based on the data sets and then determine the best fitted model to calculate the basic reproduction number *R*_0_ for A/H1N1 in China and Ebola in different regions of West Africa. The basic reproduction number *R*_0_ can be obtained from the formula *R*_0_ = exp(*rT*) [3, 4], where *r* denotes the intrinsic growth rate and *T* is the generation time of disease transmission.

Secondly, the turning point (or the inflection point of the cumulative case curve), defined as the time when the rate of case accumulation changes from increasing to decreasing (or vice versa), will be estimated for A/H1N1 in China and Ebola in different regions of West Africa. The turning point plays an important role in determining the rate of change transitions from positive to negative, that is, the moment at which the cases begin to decline. Precisely estimating this point can allow us to determine either the beginning of a new epidemic phase or the peak of the current epidemic phase, representing the point at which disease control activities take effect or the point at which an epidemic begins to wane naturally, defined by Hsieh et al. [14].

The final size of an emerging infectious disease is another important quantity for public health, which is the likely magnitude of the outbreak, and it is often called the expected final size of the epidemic [14, 15].

### 2.2. Metropolis-Hastings Algorithm and Bayesian Model Selection

The principle of MCMC methods can be briefly described as follows: build a transition kernel *k* with stationary distribution *f*(*x*) (which is a target density) and then generate a Markov chain *X* ^(*t*)^ using this kernel such that the limiting distribution of *X* ^(*t*)^ is *f*(*x*). The integral ∫_*H*_ *h*(*x*)*f*(*x*)*dx* can be approximated with the standard average (1/*T*)∑_0_^*T*^ *h*(*X* ^(*t*)^). The Metropolis-Hastings (MH) algorithm is one of the methods to realize MCMC algorithm, which can produce a Markov chain *X* ^(*t*)^ with the objective density *π*(*x*) and the transition probability *q*(·, ·). The algorithm is as follows.

Given *x* ^(*t*)^,

(1) move the chain to a new value *y* generated from the density *q*(*x* ^(*t*)^, ·).
(2) Take
  (1) $$\begin{array}{c} x^{\left(t+1\right)}=\left\{\begin{array}{cc} y, & \text{with probability }\alpha \left(x^{\left(t\right)},y\right),\\ x^{\left(t\right)}, & \text{with probability}\;\;1-\alpha \left(x^{\left(t\right)},y\right), \end{array}\right. \end{array}$$
 where
  (2) $$\begin{array}{c} \alpha \left(\;x^{\left(t\right)},y\;\right)=\min\left\{\;1,\;\;\frac{\pi \left(y\right)}{\pi \left(x^{\left(t\right)}\right)}\frac{q\left(y,x^{\left(t\right)}\right)}{q\left(x^{\left(t\right)},y\right)}\;\right\} \end{array}$$
 with
  (3) Pxt,y=qxt,y,πyqy,xt≥πxtqxt,y,qy,xtπyπxt,πyqy,xt<πxqxt,y.

The distribution *q* is called the instrumental (or proposal or candidate) distribution and probability *α*(*x* ^(*t*)^, *y*) is the Metropolis-Hastings acceptance probability [16].

Suppose that the observed data *Y* is generated by a model *g*_*j*_ ∈ *𝔐*, where *𝔐* is the finite set of competing models. Corresponding to model *g*_*j*_, there is a distinct unknown parameter vector *θ*_*j*_ of dimension *n*_*j*_ and a prior model probability *π*_*j*_ ≡ *P*(*M* = *g*_*j*_) with ∑_*g*_*j*_∈*𝔐*_ *π*_*j*_ = 1. Let Θ_*j*_ be set of all possible values for *θ*_*j*_; then *θ*_*j*_ ∈ Θ_*j*_⊆*ℝ* ^*n*_*j*_^; and let *θ* be the collection of all model-specific *θ*_*j*_'s. Now our interest lies in obtaining the posterior probabilities for the various models, *P*(*M* = *g*_*j*_∣*Y*) and then in determining the best model.

A slightly more direct (and more common) approach to estimating posterior model probabilities using MCMC has been included in the model indicator *M* as a parameter in the sampling order. As a result, most model settings require that the MCMC searches over the models and parameter space jointly. That is, the joint sampling space is

(4) $$\begin{array}{c} M\times \underset{\left(g_{j}\in M\right)}{\prod }\Theta_{j}\subset M\times \underset{\left(g_{j}\in M\right)}{\prod }R^{n_{j}}. \end{array}$$

Besides the marginal posterior model probabilities *P*(*M* = *g*_*j*_∣*Y*), this joint search also permits posterior estimation of the parameters under each model, *P*(*θ*_*j*_∣*M* = *g*_*j*_, *Y*). Assume that, corresponding to model *g*_*j*_, the likelihood function is *P*(*Y*∣*θ*_*j*_, *M* = *g*_*j*_), the prior probability is *P*(*θ*_*j*_∣*M* = *g*_*j*_), *M* is merely an indicator of which *θ*_*j*_ is relevant to *Y*, and *Y* is independent of *θ*_*j*′≠*j*_ given the model indicator *M* [17]. The following four models are employed in the present work.

Logistic model is as follows:

(5) $$\begin{array}{c} x^{\prime }\left(\;t\;\right)=rx\left(\;t\;\right)\begin{array}{c} \left(1-\frac{x\left(t\right)}{K}\right) \end{array}. \end{array}$$

Gompertz model is as follows:

(6) $$\begin{array}{c} x^{\prime }\left(\;t\;\right)=rx\left(\;t\;\right)\ln\left(\;\frac{K}{x\left(t\right)}\;\right). \end{array}$$

Rosenzweig model is as follows:

(7) $$\begin{array}{c} x^{\prime }\left(\;t\;\right)=rx\left(\;t\;\right)\left[\;{\left(\;\frac{K}{x\left(t\right)}\;\right)}^{q}-1\;\right],\;0<q\leq 1. \end{array}$$

Richards Model (the reverse Rosenzweig model) is as follows:

(8) $$\begin{array}{c} x^{\prime }\left(\;t\;\right)=rx\left(\;t\;\right)\left[\;1-{\left(\;\frac{x\left(t\right)}{K}\;\right)}^{q}\;\right],\;0<q\leq 1. \end{array}$$

For convenience, we denote, respectively, the above four models as *g*_1_, *g*_2_, *g*_3_, and *g*_4_, so *𝔐* = {*g*_1_, *g*_2_, *g*_3_, *g*_4_}. Here the positive parameter *r* denotes the intrinsic growth rate, *K* represents carry capacity, and *q* is the exponent of deviation. The above four models are widely used single species models which can be solved analytically and thus can be easily employed to fit the data and estimate the unknown parameters.

Let *N* be the unknown true cumulative number of cases with *N* = (*N*_1_,…,*N*_*n*_)′ and *Y* denote the reported cumulative cases of the emerging infectious disease with *Y* = (*Y*_1_,…,*Y*_*n*_)′. Because the reported cases having certain statistical errors are inaccurate, we assume that the reported cases follow a Poisson process. Thus, if the real cumulative number of cases at a given time *t* is *n*_*t*_, the probability of the number of cases reported is

(9) $$\begin{array}{c} P\left(\;Y_{t}=y_{t}\;|\;N_{t}=n_{t}\;\right)=\begin{array}{c} \frac{{n_{t}}^{y_{t}}}{y_{t}!}e^{-n_{t}} \end{array}. \end{array}$$

Further, we assume that the set of parameter vectors is *θ*_*j*_ = {*r*_*j*_, *K*_*j*_, *q*_*j*_}  (*j* = 1,2, 3,4), in which the parameters are independent of each other. In particular, *q*_*j*_ = 1 for models *g*_1_ and *g*_2_. For simplicity, we select noninformation prior distribution; that is, *θ*_*j*_∝ constant; thus the posterior distribution probability reads

(10) Pθj ∣ Y,M=gj∝fY ∣ θj,M=gjPθj ∣ M=gj∝∏i=1nntjytyt!e−ntj.

The step of model selection with the Metropolis-Hastings algorithm is based on a proposal for a move from model *g*_*j*_ to *g*_*j*′_, followed by acceptance or rejection of this proposal. Assume that the selection probability of model *g*_*j*_ is *π*_*j*_. The procedure given by Han and Carlin [17] is as follows:

(1) Let the initial value be *M*( = *g*_*j*_), *θ*_*j*_, *θ*_*j*′≠*j*_.
(2) Propose a new model *g*_*j*′_ with probability *q*(*g*_*j*_, ·).
(3) Accept the proposed move (from *g*_*j*_ to *g*_*j*′_) with probability
  (11) αgj,gj′=min⁡1,fY ∣ θj′,M=gj′Pθj′ ∣ M=gj′πj′qgj′,gjfY ∣ θj,M=gjPθj ∣ M=gjπjqgj,gj′.

Under the usual regularity conditions, this MH algorithm will produce samples. Provided that the sampling chain for the model indicator mixes sufficiently well, the posterior probability of model *g*_*j*_ can be estimated by

(12) $$\begin{array}{c} \hat{P}\left(\;M=g_{j}\;|\;Y\;\right)=\frac{1}{N}\overset{N}{\underset{n=1}{\sum }}I\left(\;M^{\left(n\right)}=g_{j}\;\right), \end{array}$$

which can in turn be used to estimate a Bayes factor as

(13) $$\begin{array}{c} {\hat{B}}_{j^{\prime }j}=\frac{\hat{P}\left(M=g_{j^{\prime }}\;|\;Y\right)/\hat{P}\left(M=g_{j}\;|\;Y\right)}{P\left(M=g_{j^{\prime }}\right)/P\left(M=g_{j}\right)}. \end{array}$$

The criterion of model selection based on the Bayes factor is shown in Table 1.

Based on above procedures, we realize our model selection as follows. Firstly, we obtain the Markov chains having 500000 samplers for each parameter of each model, respectively, carrying out the MCMC procedure by using an adaptive MH algorithm. Then the best model can be selected dynamically with the Markov chains of all parameters as follows.

(1) Let the initial value be *M* ^0^ = *g*_*j*_, *θ*_*j*_^0^, where *θ*_*j*_^0^ is of dimension *n*_*j*_  (*g*_*j*_ ∈ *𝔐* = {*g*_1_, *g*_2_, *g*_3_, *g*_4_}).
(2) Generate a new model *g*_*j*′_ from the discrete uniform distribution *q*(*g*_*j*_, ·), and *π*_*j*_ = (1/4)  (*j* = 1,2, 3,4), *g*_*j*′_ ∈ *𝔐*. Let *w*_*i*_ = ∑_*j*=1_^*i*^ *π*_*j*_, (*i* = 1,2, 3,4) and *w*_0_ = 0, *μ* ~ *U*(0,1); when *w*_*i*−1_ < *μ* < *w*_*i*_, let *j*′ = *i*.
(3) Repeat for *t* = 1,2 …, *N*.
(4) Evaluate the acceptance probability of the move (from *g*_*j*_ to *g*_*j*′_) by
  (14) αgj,gj′=min⁡1,fY ∣ θj′t−1,M=gj′Pθj′t−1 ∣ M=gj′πj′qgj′,gjfY ∣ θjt−1,M=gjPθjt−1 ∣ M=gjπjqgj,gj′
 with *q*(*g*_*j*_, *g*_*j*′_) = *q*(*g*_*j*′_, *g*_*j*_).
(5) Let *μ* ~ *U*(0,1), and then we have
  (15) $$\begin{array}{c} M^{\left(t\right)}=\left\{\begin{array}{cc} g_{j^{\prime }}, & \mu \leq \alpha \left(g_{j},g_{j^{\prime }}\right),\\ g_{j}, & \mu >\alpha \left(g_{j},g_{j^{\prime }}\right). \end{array}\right. \end{array}$$
(6) Return the values {*M* ^(1)^, *M* ^(2)^,…, *M* ^(*N*)^}; then we have
  (16) $$\begin{array}{c} \hat{P}\left(\;M=g_{j}\;|\;Y\;\right)=\frac{1}{N}\overset{N}{\underset{t=1}{\sum }}I\left(\;M^{\left(t\right)}=g_{j}\;\right)\;\left(g_{j}\in M\right). \end{array}$$
 The estimation of the corresponding Bayes factor is
  (17) $$\begin{array}{c} {\hat{B}}_{jj^{\prime }}=\frac{\hat{P}\left(M=g_{j^{\prime }}\;|\;Y\right)/\hat{P}\left(M=g_{j}\;|\;Y\right)}{P\left(M=g_{j^{\prime }}\right)/P\left(M=g_{j}\right)}. \end{array}$$

## 3. Validation of Model Selection Algorithm

In order to validate the proposed model selection algorithm, we generate the time series from a given model with known parameter values. To do this, we fix all parameter values of Richards model as *θ* = (*r*, *K*, *q*) = (0.3,100,0.4) and of Gompertz model as *θ* = (*r*, *K*) = (0.15,100) and the initial value *x*_0_ = 5. Solving, respectively, the two models from *t* = 1 to *t* = 40, we get forty time points of each model, denoted by *Y* = (*y*_1_,…, *y*_40_) and *Z* = (*z*_1_,…, *z*_40_) respectively.

Using the simulated data set *Y* = (*y*_1_,…, *y*_40_), we realize the model selection based on the algorithm introduced in the previous section, as shown in the first line of Table 2. Here we can calculate ${\hat{B}}_{41}=\hat{P}\left(M=4|y\right)/\hat{P}\left(M=1|y\right)=8866.4>100,{\hat{B}}_{42}$ being infinite >100 and ${\hat{B}}_{43}=144.8>100$. Thus, the evidence of selecting model *g*_4_ (Richards model) is decisive based on the criterion shown in Table 1. To further confirm the validation of the proposed method, we calculate the AIC value of each model; that is, they are 260, 241, 245, and 231 for models *g*_1_, *g*_2_, *g*_3_, and *g*_4_, respectively. The AIC value for model *g*_4_, Richards model, is the smallest, so the best model is Richards model, which is consistent with the result using Bayes factor. The estimation of parameter values for Richards model is as follows: *θ* = (*r*, *K*, *q*) = (0.3095,100.26,0.3914) being very close to the real values, shown in the third line of Table 2.

A repeat of the above procedure by using the simulated data *Z* = (*z*_1_,…, *z*_40_) gives that model *g*_2_ (Gompertz model) is decisive and is then the “best” model. The estimation of parameter values for Gompertz model is *θ* = (*r*, *K*) = (0.15041,100) which are very close to the real values, shown in the last line of Table 2.

**Figure 1:**
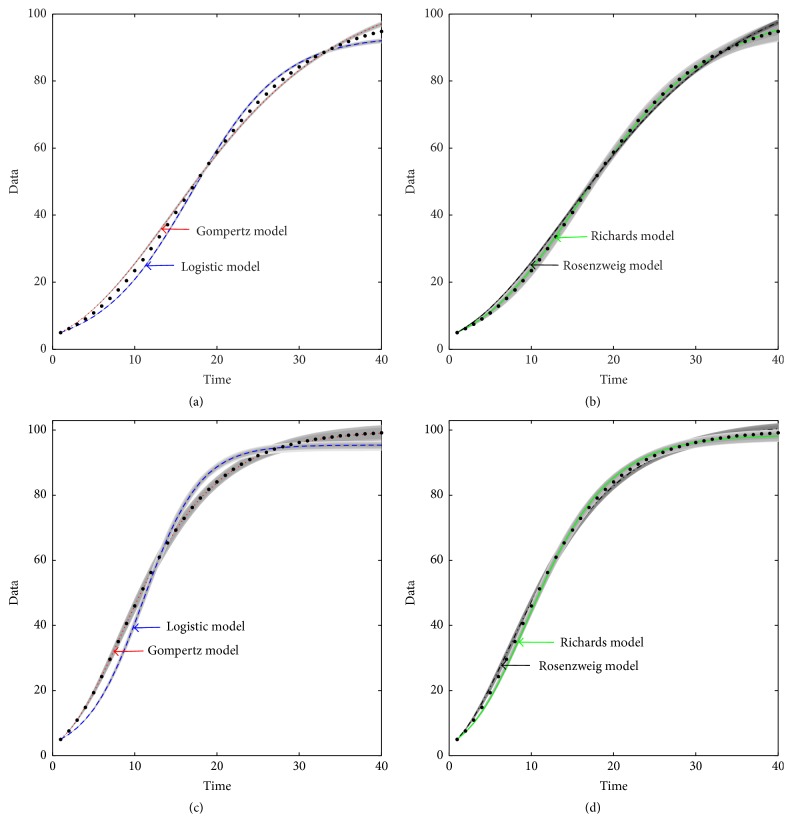
Model fitting of simulated data generated from Richards model and Gompertz model using four candidate models. The data in (a) and (b) are produced from Richards model; the data in (c) and (d) are produced from Gompertz model. The simulated data points are shown as black dot points. The curves represent the fitting to the data points for four models, respectively. The grey areas are the 95% confidence intervals of each lines.

The above results show that the proposed model selection methods based on Bayes factor and MCMC method can help us to choose the optimal model. In Figure 1, we plot the fitting results for four models based on the simulated time series generated from Richards model and Gompertz model. Although the other three models can also fit the simulated data well, it is obvious that the fitting of the Richards model and data time points is the best for time series *Y* = (*y*_1_,…, *y*_40_), as shown in Figures 1(a) and 1(b), and the fitting of the Gompertz model and data time points is the best for time series *Z* = (*z*_1_,…, *z*_40_), as shown in Figures 1(c) and 1(d).

## 4. Real Data Driven Model Selection and Results

### 4.1. A/H1N1 Data and Results

The 2009 influenza A/H1N1 pandemic outbreaks in Shaanxi Province of mainland China started from the 3rd of September. The majority of reported A/H1N1 cases were initially diagnosed in colleges and universities in early September 2009 when the universities began their fall semester and then spread to the communities in the middle of October 2009. The epidemic curve in Shaanxi Province exhibited the bimodality, where the first and small wave started around 3 September till 21 September and the second and large wave followed [11, 13]. In order to evaluate the effectiveness of control measures on A/H1N1, Tang et al. [11, 13] proposed the compartment epidemic models and then employed the A/H1N1 data sets to estimate the unknown parameters.

**Figure 2:**
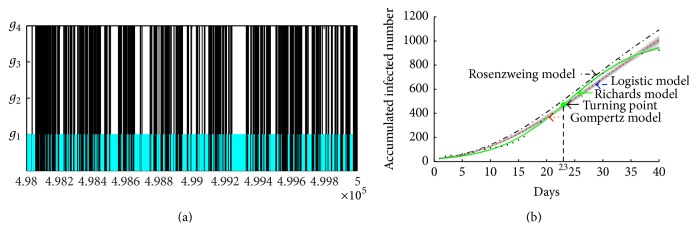
(a) Model selection based on the accumulate cases data from the 8th Hospital of Xi'an from 3 September to 21 September with the last 2000-group parameters of Markov chain; (b) model fitting of A/H1N1 data in Xi'an, 2009. The curves represent the fitting to the data for four models, respectively. The grey areas are the 95% confidence intervals of each curve. Here, cyan curve represents Logistic model; blue curve represents Gompertz model; red curve represents Rosenzweig model; black curve represents Richards model. Note that the cyan curve and black curve almost coincide.

In this subsection, we plan to realize the model selection procedures using the published accumulative cases number of A/H1N1 from the 8th Hospital of Xi'an, where the majority of the confirmed cases in the province of Shaanxi in early September 2009 were isolated. The selection results are given in the first line of Table 3 and Figure 2(a). It follows from Table 3 that Bayes factors ${\hat{B}}_{12}$, ${\hat{B}}_{13}$ are infinite (>100 naturally), which confirm that there exists decisive evidence for model *g*_1_ (i.e., Logistic model) compared with models *g*_2_ and *g*_3_. Moreover, both $1<{\hat{B}}_{14}=1.34<3$ and $1/3<{\hat{B}}_{41}=0.75<1$ mean that the selection of Logistic model and Richards model is uniform and alternating. To confirm the model selection results on A/H1N1 data set, we further calculate the AIC values which are given to be 249, 362, 592, and 254 for models *g*_1_, *g*_2_, *g*_3_, and *g*_4_, respectively. The AIC values for both Logistic model and Richards model support us to choose these two models, which are the best models for us to fit the A/H1N1 data.

To show the results of model selection intuitively, Figure 2(a) gives the selection results for the last 2000 groups of all estimated parameters from the Markov chains. In Figure 2(a), the number of four models which have been selected in the last 2000 runs is 1102, 0, 0, and 898 for models *g*_1_, *g*_2_, *g*_3_, and *g*_4_, respectively. It is easy to notice that the probabilities for Logistic model and Richards model are almost the same, which further confirm that Logistic model and Richards model are the best model for the A/H1N1 data set. Fitting, respectively, four models to the cumulate A/H1N1 case number data, we obtained model fit for the initial outbreak from September 3 to September 21, shown in Figure 2(b). It is easy to notice that the best fitted theoretical models are Logistic model and Richards model and the solution curves of Logistic model and Richards model are coincident.

The estimations of basic reproduction number *R*_0_ and turning point *t*_*i*_ are shown in the first line of Table 5 and parameters for Logistic model are shown in the first line of Table 4. For the purpose of computing *R*_0_, we employ the mean estimated generation interval of *T* = 4 days given by Tang et al. [13], which results in the estimation of *R*_0_ based on Logistic model (i.e., *R*_0_ = 1.9005 (95% CI (1.8869, 1.9142))). The likelihood-based and compartment model-based estimations of *R*_0_ are 1.663 (95% CI (1.273, 2.053)) [13] and 1.682 (95% CI (1.446, 1.918)) [11] for the period from 3 September to 21 September with a generation time of four days. All those show that in order to evaluate the emerging infectious disease we could employ the simplest model, because it allows us to identify the model parameter values more quickly and it is actually based on a small number of data points, and this is quite important for public health. The result of *t*_*i*_ = 23 for Xi'an indicates that the turning point had occurred during 25–27 September, 2009. The estimation of final size is 1013 (95% CI (996, 1030)) of the first wave, but it cannot be reached because of the beginning of the second wave.

### 4.2. Ebola Data and Results

On June 18, 2014, an Ebola outbreak emerged in Africa. The outbreak, first reported in Guinea in December, 2013, has spread to neighboring Sierra Leone and Liberia. Ebola, characterized by fever, severe diarrhea, and vomiting, has a high fatality rate, which has mooted by the World Health Organization (WHO) criteria for a serious disease. Therefore, the main propose of this subsection is to use the report data sets from the WHO about the most serious regions including Guinea, Liberia, and Sierra Leone from March 25, 2014, to May 3, 2015, in order to carry out model selections and parameters estimations and then to get the estimates of *R*_0_, turning point *t*_*i*_, and final size for Guinea, Liberia, Sierra Leone, and West Africa, respectively. Note that the sum of data from those three countries has been used for West Africa.

**Figure 3:**
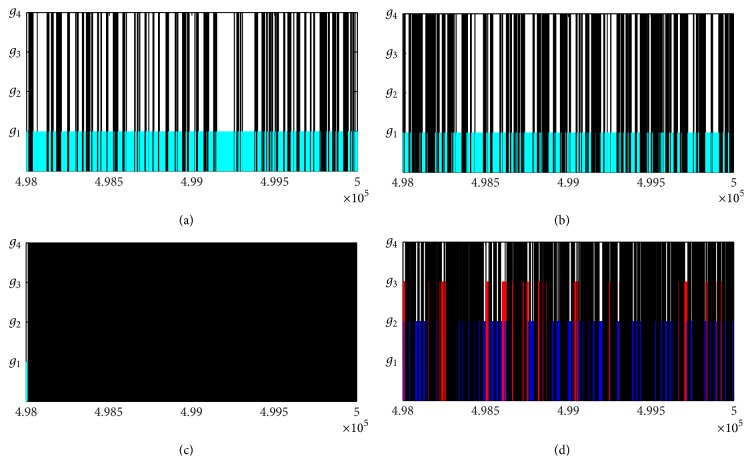
Model selection based on the accumulate Ebola cases for (a) West Africa, (b) Guinea, (c) Liberia, and (d) Sierra Leone with the last 2000-group parameters of Markov chain. The Logistic model and Richards model are selected in (a) and (b), and Richards model is selected in (c) and (d).

**Figure 4:**
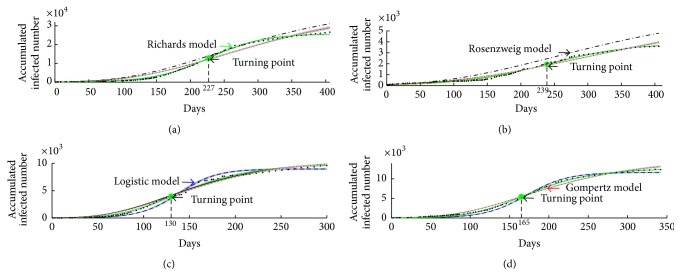
Model fitting of 2014-2015 Ebola outbreaks in (a) West Africa, (b) Guinea, (c) Liberia, and (d) Sierra Leone. Data of the cumulative numbers of infected cases are shown as black dots. The curves represent the fitting to the data for four models, respectively. The grey areas are the 95% confidence interval of each curves. Cyan curve represents Logistic model; blue curve represents Gompertz model; red curve represents Rosenzweig model; black curve represents Richards model.

**Figure 5:**
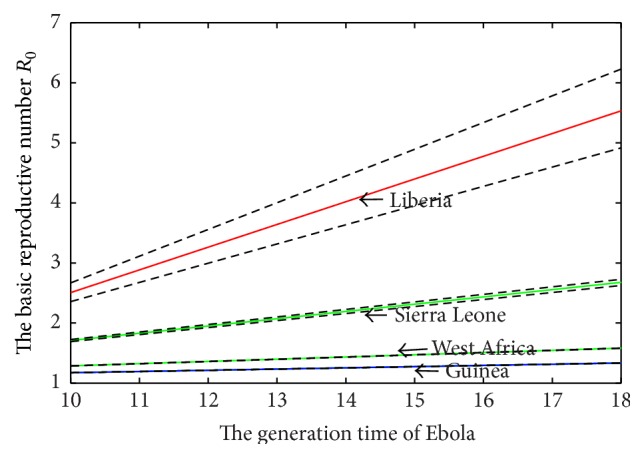
The effects of different generation time in West Africa, Sierra Leone, Liberia, and Guinea on the basic reproduction number of Ebola. Dotted lines represent the 95% confidence interval of *R*_0_ generated by the 95% confidence interval of *r*.

The selection results are shown in Table 3 and Figure 3. In Table 3, we compute the relevant Bayes factors and AICs for four candidate models. Figure 3 gives the selection result for last 2000 groups of all estimated parameters from the Markov chains based on the Ebola cases of West Africa, Guinea, Liberia, and Sierra Leone. The estimations of model parameters and *R*_0_, *t*_*i*_, and final size are shown in Tables 4 and 5, respectively. In Table 6, we compared the reported cases and model predicted cases of Ebola based on Richards model on June 14, 2015. In Figure 4, the model fitting results for four models and data sets are also provided. Note that the data points from March 25, 2014, to May 3, 2015, for West Africa and Guinea and the data points from May 27, 2014, to May 3, 2015, for Liberia and Sierra Leone have been used to fit the models. In Figure 5, we show the different *R*_0_ and relevant 95% confidence interval when the generation time changes from 10 days to 18 days.

In particular, for West Africa, the selection results are given in the second line of Table 3 and Figure 3(a). It follows from Table 3 that Bayes factors ${\hat{B}}_{12}$, ${\hat{B}}_{13}$ are infinite (>100 naturally), which indicates that there exists decisive evidence for model *g*_1_ (i.e., Logistic model) compared with models *g*_2_ and *g*_3_. Moreover, both $1<{\hat{B}}_{14}=2.1528<3$ and $1/3<{\hat{B}}_{41}=0.4645<1$ mean that the selection of Logistic model and Richards model is uniform and alternating (i.e., *q* = 1 here). To further confirm the model selection results for West Africa, the AIC values are calculated and given by 5200, 49500, 1872800, and 5400 for models *g*_1_, *g*_2_, *g*_3_, and *g*_4_, respectively. The AIC values for both Logistic model and Richards model further indicate that these two models are the best. In Figure 3(a), the numbers of four models which have been selected in the last 2000 runs are 1168, 0, 0, and 832 for models *g*_1_, *g*_2_, *g*_3_, and *g*_4_, respectively. It is interesting to notice that the probability for Logistic model is the biggest one and Richards model is the second one, which further confirms that Logistic model is the best model for West Africa data set. In Figure 4(a), it is easy to notice that the best fitted theoretical models are Logistic model and Richards model and the solution curves of Logistic model and Richards model are almost coincident.

The estimations of the parameters for Logistic model are shown in the second line of Table 4 and basic reproduction number *R*_0_ and turning point *t*_*i*_ based on West Africa data are shown in the second line of Table 5. The mean estimated generation interval *T* = 12 days given by Chowell and Nishiura [12] is used to calculate the basic reproduction number *R*_0_. Based on Logistic model *R*_0_ is estimated to be 1.3522 (95% CI (1.3506, 1.3537)) and the variation in *R*_0_ with different generation intervals *T* is shown in Figure 5. The turning point is *t*_*i*_ = 227 (95% CI (226, 228)) which indicates that the turning point had occurred during 6–8 November, 2014, for West Africa. The estimation of final size is 25794 (95% CI (25630, 25958)) which could have occurred during 13–17 September, 2015. On June 14, 2015, the reported cases are 26742 and the model predicted cases are 25693 and the rate of underestimated rate of model is −5.9%.

For Guinea, the selection results are given in the third line of Table 3 and Figure 3(b). From Table 3, Bayes factors ${\hat{B}}_{12}$, ${\hat{B}}_{13}$ are infinite (>100 naturally), which confirm that there exists decisive evidence for model *g*_1_ (i.e., Logistic model) compared with models *g*_2_ and *g*_3_. Moreover, both $1<{\hat{B}}_{14}=1.25<3$ and $1/3<{\hat{B}}_{41}=0.8<1$ mean that the selection of the Logistic model and the Richards model is uniform and alternating. The AIC values are 1991, 3427, 18476, and 1998 for models *g*_1_, *g*_2_, *g*_3_, and *g*_4_, respectively. The AIC values for both Logistic model and Richards model further indicate that these two models are the best for us to fit Guinea data. In Figure 3(b), the numbers of selected about four models which have been selected in the last 2000 runs are 1028, 0, 0, and 972 for models *g*_1_, *g*_2_, *g*_3_, and *g*_4_, respectively, which further shows that the Logistic model and the Richards model are the best model for Guinea data set, as shown In Figure 4(b). Compared with the selection results for West Africa we conclude that the outbreak pattern of West Africa follows Guinea.

The estimations of the parameters for Logistic model are shown in the third line of Table 4 and basic reproduction number *R*_0_ and turning point *t*_*i*_ based on Guinea data are shown in the third line of Table 5. The estimation of *R*_0_ based on Logistic model is 1.2101 (95% CI (1.2084, 1.2119)) with generation interval *T* = 12 days and the different estimations with different *T* are shown in Figure 5. The result of *t*_*i*_ = 239 (95% CI (237, 241)) indicates that the turning point had occurred during 15–19 November, 2014. The estimation of final size is 3916 (95% CI (3865, 3967)) which could have occurred during 24–31 December, 2015. On June 14, 2015, the reported cases are 3674 and the model predicted cases are 3778 and the rate of overestimated model is +2.8%.

For Liberia, the selection results are given in the fourth line of Table 3 and Figure 3(c). It follows from Table 3 that Bayes factors ${\hat{B}}_{42}$, ${\hat{B}}_{43}$ are infinite (>100 naturally), which suggests that there exists decisive evidence for model *g*_4_ (i.e., Richards model) compared with models *g*_2_ and *g*_3_. Moreover, ${\hat{B}}_{14}<1/100$ and ${\hat{B}}_{41}=500000>100$ indicate that the evidence for the selection of Richards model is decisive. Meanwhile, we calculate the AIC values which are given by 6308, 6547, 7980, and 2559 for models *g*_1_, *g*_2_, *g*_3_, and *g*_4_, respectively. It supports us to choose Richards model, which is the best model for us to fit Liberia data. In Figure 3(c), the numbers of four models which have been selected in the last 2000 runs are 1, 0, 0, and 1999 for models *g*_1_, *g*_2_, *g*_3_, and *g*_4_, respectively, which further confirms that Richards model is the best model for Liberia data set, as shown in Figure 4(c).

The estimation of *R*_0_ based on Logistic model is 3.0234 (95% CI (2.6063, 3.4881)), shown in Table 5 with *T* = 12 days, and variation in *R*_0_ with different values of *T* is shown in Figure 5. The result of *t*_*i*_ = 130 (95% CI (121, 149)) indicates that the turning point occurred during 23 September–21 October, 2014. The estimation of final size is 3916 (95% CI (3865, 3967)) which occurred during 24–31 December, 2015. On June 14, 2015, the reported cases are 10666 and the model predicted cases are 9843 and the rate of underestimated model is −7.7%.

For Sierra Leone, it follows from Table 3 that Bayes factors ${\hat{B}}_{21}=102310>100$, ${\hat{B}}_{31}=34750>100$, and ${\hat{B}}_{41}=362940>100$, which implies that there exists decisive evidence for models *g*_2_, *g*_3_, and *g*_4_ compared with model *g*_1_ (i.e., Logistic model). Moreover, both $3<{\hat{B}}_{42}=3.55<10$ and $10<{\hat{B}}_{43}=10.48<30$ mean that the evidence for the selection of Richards model is stronger than model *g*_3_ and more substantial than model *g*_2_. To further confirm the model selection results, we calculate the AIC values to be 15432, 6251, 7038, and 5400 for models *g*_1_, *g*_2_, *g*_3_, and *g*_4_, respectively. The AIC value for Richards model supports us to choose Richards model, which is the best model for us to fit Sierra Leone data. In Figure 3(d), the numbers of four models which have been selected in the last 2000 runs are 2, 408, 205, and 1385 for models *g*_1_, *g*_2_, *g*_3_, and *g*_4_, respectively, which further confirm that Richards model is the best model for Sierra Leone data set, as shown in Figure 4(d).

The estimation of *R*_0_ based on Richards model is 1.9018 (95% CI (1.8565, 1.9478)) with generation interval *T* = 12 days and variation in *R*_0_ with different values of *T* is shown in Figure 5. The result of *t*_*i*_ = 165 (95% CI (157, 174)) indicates that the turning point had occurred during 27 October–12 November, 2014. The estimation of final size is 12633 (95% CI (12515, 12750)) which could have occurred during 15–22 December, 2015. On June 14, 2015, the reported cases are 12965 and the model predicted cases are 12515 and the rate of underestimated model is −3.5%.

Comparing the actual reported cases and the model predicted cases, on 14 June, 2015, the rates of underestimated or overestimated model are, respectively, −5.9%, +2.8%, −7.7%, and −3.5% for West Africa, Guinea, Liberia, and Sierra Leone, as shown in Table 6. In Liberia, the underestimated rate is bigger than others because the data had changed due to ongoing reclassification, retrospective investigation, and availability of laboratory results, and the data of Liberia had significant adjustments. This also is why *R*_0_ for Liberia is the biggest. Note that the reported accumulated cases including confirmed, probable, and suspected cases in Liberia have been revised largely; for example, there are 4665, 6535, and 6525 cases on October 23, October 27, and November 2, 2014, respectively. Similarly, the reported accumulated cases in Sierra Leone are 3896, 5235, and 4759 on October 23, October 27, and November 2, 2014, respectively. Those big differences could result in the big variances in estimating and predicting the outbreaks of Ebola in West Africa. Therefore, the more precise data sets are, the more accurate estimation and predication are.

## 5. Discussion and Conclusions

On the basis of four simplest single species models, the model selection, and MCMC method we choose the best model to fit the A/H1N1 data set in China and Ebola data sets in West Africa. This allows us to estimate the basic reproduction number, the turning point, and final size more quickly and accurately for the emerging infectious disease compared with some complex compartment models.

Our estimate of *R*_0_ = 1.9005 with (95% CI (1.8869, 1.9142)) on A/H1N1 is quite similar to that from the data for Shaanxi Province obtained by Tang et al. [11, 13] but with little differences that could well be associated with differences in methodology. Further, many factors such as differences in population densities, realization of control measure, and mobility of the population among regions led to a wide range of reproduction number. Our estimated reproduction numbers from the hospital notifications are in broad agreement with those obtained in studies on data from Mexico (95% CI (1.2, 1.6)) [18], the United States of America (95% CI (1.7, 1.8)) [19], and New Zealand (95% CI (1.80, 2.15)) [20]. Thus, we believe that the best model (i.e., Richards model) can be used for rapid epidemic modeling in the face of public health crisis.

When we fit the data sets for Ebola cases in West Africa, the selection of the most appropriate model is Logistic model or Richards model. Reproductive numbers *R*_0_ are 1.3522 (95% CI (1.3506, 1.3537)) for West Africa, 3.0234 (95% CI (2.6063, 3.4881)) for Liberia, 1.2101 (95% CI (1.2084, 1.2119)) for Guinea, and 1.9018 (95% CI (1.8565, 1.9478)) for Sierra Leone. Using early phase of Ebola outbreaks in West Africa 2014, Chowell and Nishiura [12] estimated *R*_0_ for those three countries, which were given by 1.96 (95% CI (1.92, 2.01)) for Liberia and 3.07 (95% CI (2.85, 3.32)) for Sierra Leone. Althaus [21] formulated a susceptible-exposed-infectious-removal (SEIR) model and employed the data from March 22, 2014, to August 20, 2014, to get the maximum likelihood estimates of *R*_0_, where *R*_0_ = 1.51 (95% CI (1.50, 1.52)) for Guinea, 2.53 (95% CI (2.41, 2.67)) for Sierra Leone, and 1.59 (95% CI (1.57, 1.60)) for Liberia. WHO Ebola Response Team [22] employed the data by September 14, 2014, and got *R*_0_ = 1.51 (95% CI (1.41, 1.60)) for Liberia, 1.81 (95% CI (1.60, 2.03)) for Guinea, and 1.38 (95% CI (1.27, 1.51)) for Sierra Leone. It is worth noticing that the estimations of *R*_0_ by using data points in different periods are quite different, and any differences could well be associated with variations in methodology and differences at times or at stages.

For the previous Ebola outbreaks in Central Africa, Chowell et al. [23] developed a homogenous mixing SEIR model and got *R*_0_ = 1.83 for Congo in 1995 and 1.34 for Uganda in 2000. However, the estimations of *R*_0_ in the present paper show that *R*_0_ for Liberia is the biggest, followed for Sierra Leone, and the smallest is for Guinea. As mentioned before, the main reason why *R*_0_ for Liberia is bigger than others is that the ongoing reclassification, retrospective investigation, and availability of laboratory results make the data of Liberia having significant adjustment. Moreover, the suspected cases were increased significantly, while the confirmed cases were increased slowly related to the suspected cases.

The turning point and final size have been also estimated and calculated. For example, the turning point for West Africa was 227 days which corresponds to 5 November, 2014, and the turning points for Guinea, Liberia, and Sierra Leone were about October or November, 2014. Further, the final breakout time will be September or December, 2015, with final size of 3916 (95% CI (3865, 3967)) for Guinea, 9886 (95% CI (9740, 10031)) for Liberia, 12633 (95% CI (12515, 12750)) for Sierra Leone, and 25794 (95% CI (25630, 25958)) for West Africa, respectively, as shown in Table 3. Note that the Ebola outbreak in Liberia was declared over on 9 May, after 42 complete days that elapsed since the burial of the last confirmed case, but the estimation of final time of Liberia was September 23, 2015, because of the accumulative reported numbers of suspected cases being increasing. That is the reason of the country having entered a 3-month period of heightened vigilance from May 9, 2015, and WHO will maintain an enhanced presence in the country until the end of 2015, with a particular focus on areas that border Guinea and Sierra Leone (http://apps.who.int/ebola/en/current-situation/ebola-situation-report-13-may-2015).

For the results of model selection, the most appropriate model is Logistic model or Richards model which requires only cumulative case data from an epidemic curve (Table 7). Note that for the earlier stages of an epidemic such as Ebola in Guinea the Logistic model cannot fit the data well [12]. However, our main results show that the Logistic model could be a candidate to fit the data with more time points. All those indicate that the model selection depends on the length of the time series. Moreover, Logistic model is a special case of Richards model with the exponent of deviation parameter 1. Therefore, we conclude that Richards model could be chosen firstly when estimating *R*_0_ that require more extensive and detailed data [24, 25]. E. Tjørve and K. M. C. Tjørve [26] indicated that Gompertz model is also a special case of Richards model, but our results indicate that Gompertz model may not be a suitable candidate for describing the data of emerging infectious diseases.

**Figure 6:**
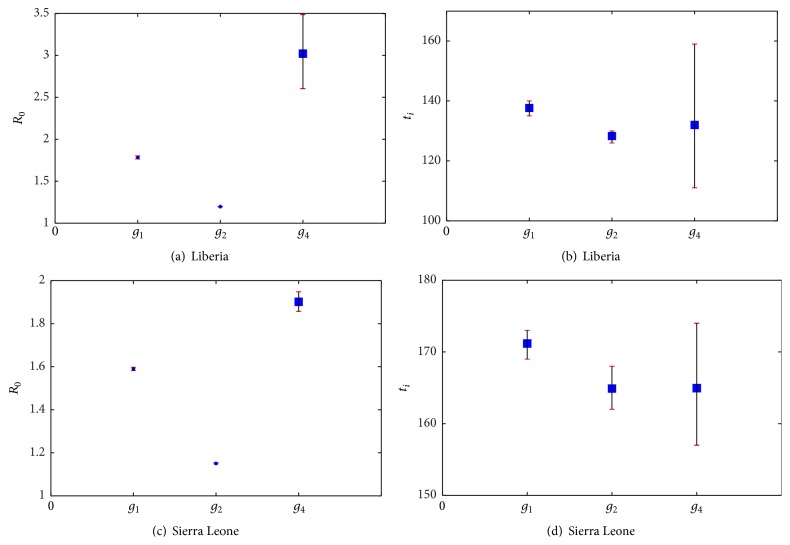
The estimation of *R*_0_ and turning point *t*_*i*_. Here the 95% CI and the deviation between estimated values and correct values of Logistic model, Richards model, and Gompertz model for Sierra Leone and Liberia dataset have been given.

In Figure 4, we fit the data sets for Guinea, Liberia, Sierra Leone, and West Africa based on four candidate models, and our results show that the best models are different for different data sets. In particular, the “best” model is Richards model for Liberia and Sierra Leone, and *R*_0_ could be underestimated if we choose the Logistic model for Liberia and Sierra Leone, while turning point *t*_*i*_ could be underestimated if we choose Gompertz model for Liberia and Sierra Leone, as shown in Table 8 and Figure 6. The error is too big when fitting data of Liberia and Sierra Leone with Rosenzweig model, so we only compare the estimation of *R*_0_ and *t*_*i*_ about Logistic, Gompertz, and Richards model.

By the analysis of Ebola data, we get that model selection uncertainty caused a magnification of the standard error of the estimation of *R*_0_ and *t*_*i*_, so model selection is necessary when fitting specific data with model. That is to say adopting the bad model would probably cause overestimation or underestimation of parameters, basic reproduction number, and final size. Thus, it has to be emphasized that the model selection is essential for investigating dynamic of the emerging infectious disease based on the available data set and arbitrarily picking a model without any consideration of alternatives is inadvisable.

## Figures and Tables

**Table 1:** Evidence categories for the Bayes factor (Jeffreys, 1961 [10]).

Bayes factor	Interpretation
*B*_*j*′*j*_ < 1/100	Decisive evidence for model *j*
1/100 < *B*_*j*′*j*_ < 1/30	Very strong evidence for model *j*
1/30 < *B*_*j*′*j*_ < 1/10	Strong evidence for model *j*
1/10 < *B*_*j*′*j*_ < 1/3	Substantial evidence for model *j*
1/3 < *B*_*j*′*j*_ < 1	Anecdotal evidence for model *j*
*B*_*j*′*j*_ = 1	No evidence
1 < *B*_*j*′*j*_ < 3	Anecdotal evidence for model *j*′
3 < *B*_*j*′*j*_ < 10	Substantial evidence for model *j*′
10 < *B*_*j*′*j*_ < 30	Strong evidence for model *j*′
30 < *B*_*j*′*j*_ < 100	Very strong evidence for model *j*′
*B*_*j*′*j*_ > 100	Decisive evidence for model *j*′

**(a):** 

Data source	*j*′	*j*			
		Logistic	Gompertz	Rosenzweig	Richards
Richards model	Logistic	1	—	—	—
	Gompertz	Inf	1	Inf	—
	Rosenzweig	Inf	—	1	—
	Richards model	8866.4	Inf	144.8	1
	AIC	260	241	245	231

Gompertz model	Logistic	1	—	—	—
	Gompertz	Inf	1	19.8855	13.0241
	Rosenzweig	Inf	—	1	—
	Richards model	Inf	—	Inf	1
	AIC	618	440	601	556

**(b):** 

Data source	Parameter	Mean	Std.	MC_err	Tau	Geweke
Richards model	*r*	0.3095	0.0335	3.9372*e* − 04	95.36	0.9923
	*K*	100.26	2.8313	0.0255	85.563	0.9991
	*q*	0.3914	0.0579	6.7027*e* − 04	95.533	0.9919

Gompertz model	*r*	0.1504	0.0062	9.2746*e* − 05	60.727	0.9983
	*K*	100	1.8169	0.0249	62.058	0.9991

— means a very small number.

Inf indicates a sufficiently big number.

**Table 3:** The corresponding Bayes factor ${\hat{B}}_{j^{\prime }j}=\hat{P}(m=j^{\prime }|y)/\hat{P}(M=j|y)$ and AIC about four models based on A/H1N1 and Ebola data.

Data source	*j*′	*j*			
		Logistic	Gompertz	Rosenzweig	Richards
H1N1	Logistic	1	Inf	Inf	1.34
	Gompertz^0^	0	1	/	—
	Rosenzweig^0^	0	/	1	—
	Richards model	0.75	Inf	Inf	1
	AIC	249	362	592	254

West Africa	Logistic	1	Inf	Inf	2.1528
	Gompertz^0^	0	1	/	—
	Rosenzweig^0^	0	/	1	—
	Richards model	0.4645	Inf	Inf	1
	AIC	5200	49500	1872800	5400

Guinea	Logistic	1	Inf	Inf	1.25
	Gompertz^0^	0	1	/	—
	Rosenzweig^0^	0	/	1	—
	Richards model	0.8	Inf	Inf	1
	AIC	1991	3427	18476	1998

Liberia	Logistic	1	Inf	Inf	—
	Gompertz^0^	0	1	/	—
	Rosenzweig^0^	0	/	1	—
	Richards model	5*∗*10^5^	Inf	Inf	1
	AIC	6308	6547	7980	2559

Sierra Leona	Logistic	1	—	—	—
	Gompertz	102310	1	2.96	0.28
	Rosenzweig	34750	0.34	1	0.095
	Richards model	362940	3.55	10.48	1
	AIC	15432	6251	7038	5400

— means a very small number.

Inf means a sufficiently big number.

0 means the probability of being chosen for model is zero.

/ means a no number (*i*.*e.*, 0/0).

**Table 4:** The estimations of all parameters with respect to the best model.

Data source	Parameter	Mean	Std.	MC_err	Tau	Geweke
H1N1	*r*	0.1605	9.1570*e* − 04	3.3003*e* − 06	6.6007	0.9999
	*K*	1013	8.6356	0.0341	6.6724	0.9997

West Africa	*r*	0.0251	4.8634*e* − 05	1.5757*e* − 07	6.6144	0.9999
	*K*	25794	83.712	0.2631	6.6658	0.9999

Guinea	*r*	0.0159	6.1289*e* − 05	2.1429*e* − 07	6.7164	0.9999
	*K*	3916	26.131	0.1143	6.6456	0.9999

Liberia	*r*	0.0919	6.19*e* − 03	6.1022*e* − 05	44.625	0.9973
	*K*	9886	74.03	0.5253	29.833	0.9999
	*q*	0.2333	0.0225	2.0514*e* − 04	39.52	0.9963

Sierra Leona	*r*	0.0536	1.0186*e* − 03	4.0645*e* − 06	12.058	0.9999
	*K*	12633	59.697	0.2866	11.61	0.9999
	*q*	0.3985	0.0149	6.4121*e* − 05	12.063	0.9997

**Table 5:** The estimations of *R*_0_, turning point *t*_*i*_, and final size for the best model.

Data source	*R*_0_	95% CI	*t*_*i*_	95% CI	Final size	95% CI
H1N1	1.9005	(1.8869, 1.9142)	23^1^	(22, 24)	1013^*∗*^	(996, 1030)
West Africa	1.3522	(1.3506, 1.3537)	227^2^	(226, 228)	25794^3^	(25630, 25958)
Guinea	1.2101	(1.2084, 1.2119)	239^4^	(237, 241)	3916^5^	(3865, 3967)
Liberia	3.0234	(2.6063, 3.4881)	130^6^	(121, 149)	9886^7^	(9740, 10031)
Sierra Leona	1.9018	(1.8565, 1.9478)	165^8^	(157, 174)	12633^9^	(12515, 12750)

^1^Denoting turning point during Sep. 25–Sep. 27, 2009.

^2^Denoting turning point during Nov. 6–Nov. 8, 2014.

^3^Denoting the final time during Sep. 13–Sep. 17, 2015.

^4^Denoting turning point during Nov. 15–Nov. 19, 2014.

^5^Denoting the final time during Dec. 24–Dec. 31, 2015.

^6^Denoting turning point during Sep. 23–Oct. 21, 2014.

^7^Denoting the final time during Sep. 19–Sep. 26, 2015.

^8^Denoting turning point during Oct. 27–Nov. 12, 2014.

^9^Denoting the final time during Dec. 15–Dec. 22, 2015.

^*∗*^the first stage cannot reach final size because of the beginning of the second stage.

*R*_0_ was computed using the mean generation interval of *T* = 4 days [11] about A/H1N1 and *T* = 12 days [12] about Ebola.

**Table 6:** Comparison of the reported and model predicted cases of Ebola based in Richards model on June 14, 2015.

Source of data	Reported cases (number)^†^	Predicted cases (number)	The rate of underestimated or overestimated model
West Africa	27305	25693	−5.9%
Guinea	3674	3778	+2.8%
Liberia	10666	9842	−7.7%
Sierra Leone	12965	12515	−3.5%

^†^Source: World Health Organization (http://apps.who.int/ebola/current-situation/ebola-situation-report-17-june-2015).

**Table 7:** The selection of model about different data.

Data	Xi'an	West Africa	Guinea	Liberia	Sierra Leone
	(H1N1)	(Ebola)	(Ebola)	(Ebola)	(Ebola)
Model	L (R)	L (R)	L (R)	R	R

L denotes Logistic model.

R represents Richards model.

L (R) means both Logistic model and Richards model.

**Table 8:** The estimation of *R*_0_ and turning point *t*_*i*_, 95% conditional confidence (95% CI) for each dataset and candidate model.

Model	*R*_0_	95% CI	*t*_*i*_	95% CI
Liberia (Ebola)				
*g*_4_ Richards model	3.0223	(2.6026, 3.4855)	130	(111, 159)
*g*_1_ Logistic model	1.784	(1.7661, 1.802)	137	(135, 140)
*g*_2_ Gompertz model	1.197	(1.1935, 1.2005)	128	(126, 130)

Sierra Leone (Ebola)				
*g*_4_ Richards model	1.9016	(1.8563, 1.9475)	165	(157, 174)
*g*_2_ Gompertz model	1.1507	(1.1478, 1.1536)	164	(162, 168)
*g*_1_ Logistic model	1.5894	(1.582, 1.5967)	171	(169, 173)

The models are sorted from the best to the worst.

## References

[1] Lipsitch M., Cohen T., Cooper B. (2003). Transmission dynamics and control of severe acute respiratory syndrome. *Science*.

[2] Riley S., Fraser C., Donnelly C. A. (2003). Transmission dynamics of the etiological agent of SARS in Hong Kong: impact of public health interventions. *Science*.

[3] Hsieh Y.-H., Ma S., Velasco Hernandez J. X., Lee V. J., Lim W. Y. (2011). Early outbreak of 2009 influenza a (H1N1) in mexico prior to identification of pH1N1 virus. *PLoS ONE*.

[4] Hsieh Y. H., Lee J.-Y., Chang H. L. (2004). SARS epidemiology, logistic-type model, and cumulative case number. *Emerging Infectious Diseases*.

[5] Hsieh Y.-H., Ma S. (2009). Intervention measures, turning point, and reproduction number for dengue, Singapore. *The American Journal of Tropical Medicine and Hygiene*.

[6] Akaike H. (1981). Likelihood of a model and information criteria. *Journal of Econometrics*.

[7] Burnham K. P., Anderson D. R. (2002). *Model Selection and Multimodel Inference: A Practical Information-Theoretic Approach*.

[8] McQuarrie A. D. R., Tsai C.-L. (1998). *Regression and Time Series Model Selection*.

[9] Posada D., Buckley T. R. (2004). Model selection and model averaging in phylogenetics: advantages of Akaike Information Criterion and Bayesian approaches over likelihood ratio tests. *Systematic Biology*.

[10] Jeffreys D. (1961). *Theory of Probability*.

[11] Tang S. Y., Xiao Y. N., Yang Y. P., Zhou Y., Wu J., Ma Z. (2010). Community-based measures for mitigating the 2009 H1N1 pandemic in China. *PLoS ONE*.

[12] Chowell G., Nishiura H. (2014). Transmission dynamics and control of Ebola virus disease (EVD): a review. *BMC Medicine*.

[13] Tang S. Y., Xiao Y. N., Yuan L., Cheke R. A., Wu J. (2012). Campus quarantine (*Fengxiao*) for curbing emergent infectious diseases: lessons from mitigating A/H1N1 in Xi'an, China. *Journal of Theoretical Biology*.

[14] Hsieh Y.-H., Fisman D. N., Wu J. H. (2010). On epidemic modeling in real time: an application to the 2009 Novel A (H1N1) influenza outbreak in Canada. *BMC Research Notes*.

[15] Ma J. L., Earn D. J. E. (2006). Generality of the final size formula for an epidemic of a newly invading infectious disease. *Bulletin of Mathematical Biology*.

[16] Robert C. P., Casella G. (2010). *Introducing Monte Carlo Methods with R*.

[17] Han C., Carlin B. P. (2001). Markov Chain Monte Carlo Methods for computing Bayes factors: a comparative review. *Journal of the American Statistical Association*.

[18] Fraser C., Donnelly C. A., Cauchemez S. (2009). Pandemic potential of a strain of influenza A (H1N1): early findings. *Science*.

[19] White L. F., Wallinga J., Finelli L. (2009). Estimation of the reproductive number and the serial interval in early phase of the 2009 influenza A/H1N1 pandemic in the USA. *Influenza and Other Respiratory Viruses*.

[20] Nishiura H., Wilson N., Baker M. G. (2009). Estimating the reproduction number of the novel influenza A virus (H1N1) in a Southern Hemisphere setting: preliminary estimate in New Zealand. *The New Zealand Medical Journal*.

[21] Althaus C. L. (2014). Estimating the reproduction number of Zaire ebolavirus (EBOV) during the 2014 outbreak in West Africa. *PLoS Currents Outbreaks*.

[22] WHO Ebola Response Team (2014). Ebola virus disease in West Africa—the first 9 months of the epidemic and forward projections. *The New England Journal of Medicine*.

[23] Chowell G., Hengartner N. W., Castillo-Chavez C., Fenimore P. W., Hyman J. M. (2004). The basic reproductive number of Ebola and the effects of public health measures: the cases of Congo and Uganda. *Journal of Theoretical Biology*.

[24] Wallinga J., Lipsitch M. (2007). How generation intervals shape the relationship between growth rates and reproductive numbers. *Proceedings—Biological Sciences*.

[25] Chowell G., Miller M. A., Viboud C. (2008). Seasonal influenza in the United States, France, and Australia: transmission and prospects for control. *Epidemiology and Infection*.

[26] Tjørve E., Tjørve K. M. C. (2010). A unified approach to the Richards-model family for use in growth analyses: why we need only two model forms. *Journal of Theoretical Biology*.

